# A bibliometric analysis of myocardial ischemia/reperfusion injury from 2000 to 2023

**DOI:** 10.3389/fcvm.2023.1180792

**Published:** 2023-06-13

**Authors:** Yifei Wang, Lijun Guo, Zhibo Zhang, Shuangqing Fu, Pingping Huang, Anzhu Wang, Mi Liu, Xiaochang Ma

**Affiliations:** ^1^ Xiyuan Hospital, China Academy of Chinese Medical Sciences, Beijing, China; ^2^ Graduate School, Beijing University of Chinese Medicine, Beijing, China; ^3^ National Clinical Research Center for Chinese Medicine Cardiology, Beijing, China

**Keywords:** myocardial ischemia/reperfusion injury, reperfusion therapy, myocardial infarction, mechanism, multi-target therapy, bibliometric analysis

## Abstract

**Background:**

Myocardial ischemia/reperfusion injury (MIRI) refers to the more severe damage that occurs in the previously ischemic myocardium after a short-term interruption of myocardial blood supply followed by restoration of blood flow within a certain period of time. MIRI has become a major challenge affecting the therapeutic efficacy of cardiovascular surgery.

**Methods:**

A scientific literature search on MIRI-related papers published from 2000 to 2023 in the Web of Science Core Collection database was conducted. VOSviewer was used for bibliometric analysis to understand the scientific development and research hotspots in this field.

**Results:**

A total of 5,595 papers from 81 countries/regions, 3,840 research institutions, and 26,202 authors were included. China published the most papers, but the United States had the most significant influence. Harvard University was the leading research institution, and influential authors included Lefer David J., Hausenloy Derek J., Yellon Derek M., and others. All keywords can be divided into four different directions: risk factors, poor prognosis, mechanisms and cardioprotection.

**Conclusion:**

Research on MIRI is flourishing. It is necessary to conduct an in-depth investigation of the interaction between different mechanisms and multi-target therapy will be the focus and hotspot of MIRI research in the future.

## Introduction

The heart is a vital organ in maintaining the body's circulatory system, and the heart muscle requires an adequate supply of blood and oxygen to maintain its function. Myocardial infarction (MI) is a consequence of coronary artery occlusion, resulting in irreversible damage to the myocardium due to ischemia and hypoxia, and poses a severe threat to human health, with high rates of disability and mortality worldwide (1–3).

As early as the 1970s, Ginks et al. (4) performed myocardial ischemia-reperfusion mapping in dogs and found that reperfusion therapy was effective in restoring blood flow and reducing myocardial injury after MI. After years of research and observation, reperfusion therapy such as percutaneous coronary intervention (PCI) and coronary artery bypass grafting (CABG) have become the first-line treatment strategy for MI (5, 6). However, it has also been found that these therapies may induce myocardial, vascular, or electrophysiological dysfunction, leading to worsened cardiac function, and is responsible for up to 50% of the final infarct size. This phenomenon is known as myocardial ischemia/reperfusion injury (MIRI), which reduces the efficacy of myocardial reperfusion therapy (7–10).

Although more effective reperfusion techniques and drugs that improve MIRI have emerged, the incidence of secondary myocardial damage after blood flow restoration remains high due to narrow intervention windows and individual differences in susceptibility to reperfusion injury (11, 12). Therefore, the pathogenesis and prevention of MIRI remain a research hotspot in the cardiovascular field.

Bibliometrics presents the knowledge structure and frontier trends of a research field by modern techniques to visualize countries, institutions, authors, journals, documents and keywords (13, 14). Therefore, we reviewed the literature on Myocardial Ischemia-Reperfusion in the Web of Science Core Collection (WoSCC) database to provide a reference for future research on Myocardial Ischemia-Reperfusion.

## Methods

### Data sources

The data for this study was obtained from WoSCC. In order to more accurately capture the topic, we conducted subject searches in SCI-Expanded, SSCI, CCR-Expanded, IC, and ESCI. The search formula for this study was set as follows: TS = (myocardial NEAR/1 “reperfusion injur*”) OR TS = (Cardiac NEAR/1 “reperfusion injur*”). The search was conducted from January 1, 2000 to January 7, 2023, with article and review types selected, and English language limited. A total of 5,595 papers were obtained. The results were exported in *txt*. format as “Full Record and Cited References”. To prevent data deviation due to database updates, the data search and export were completed on January 7, 2023.

### Analysis method

We primarily employed VOSviewer for data visualization, in conjunction with Excel, CiteSpace 6.1.R6, and Pajek 5.16 (15, 16). Firsty import the data into the CiteSpace software and check the duplicate data. Second, Synonyms are modified and merged before each visualization to show results more accurately. Third, we selected an appropriate number of nodes and set corresponding “Layout” parameters in VOSviewer, while leaving other options at their default values. We select the appropriate number of nodes for data visualization.

## Results

### Analysis of annual publications distribution

**Figure 1:**
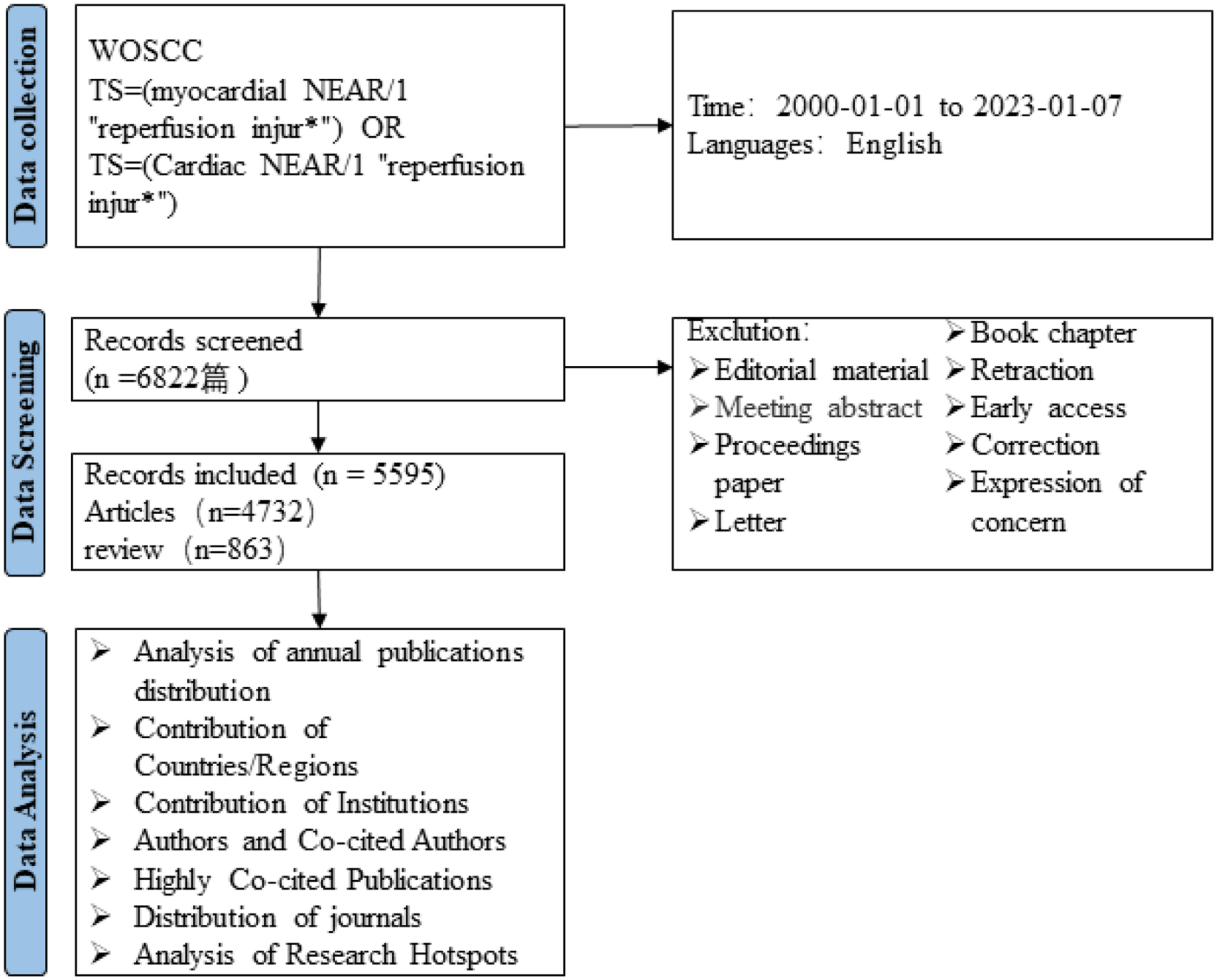
Flowchart of literature selection.

**Figure 2:**
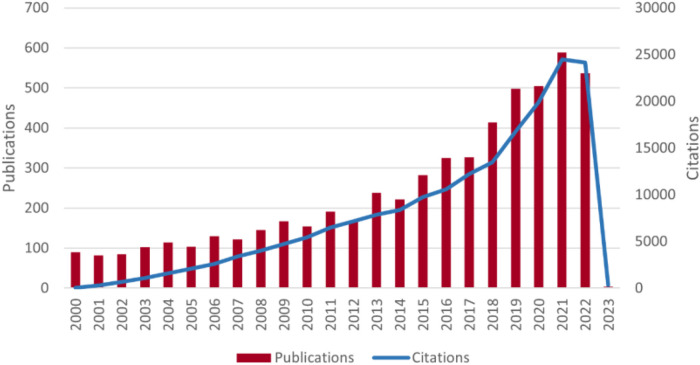
Trends in the growth of publications and the number of cited papers worldwide from 2000 to 2023. The data for 2023 are not complete.

According to the search results, a total of 5,595 papers related to MIRI were collected by WoSCC from January 1st, 2000 to January 7th, 2023, as shown in Figure 1. Among them, there were 4,732 articles and 863 reviews, with a total citation count of 160,289 (excluding self-citations), an average citation frequency of 33.51 times per article, and an h-index of 165. Figure 2 shows the annual publication and citation volume. Overall, research related to MIRI showed an increasing trend, with the highest number of publications (589) and citations (24,492) in 2021.

### Contribution of countries/regions

**Figure 3:**
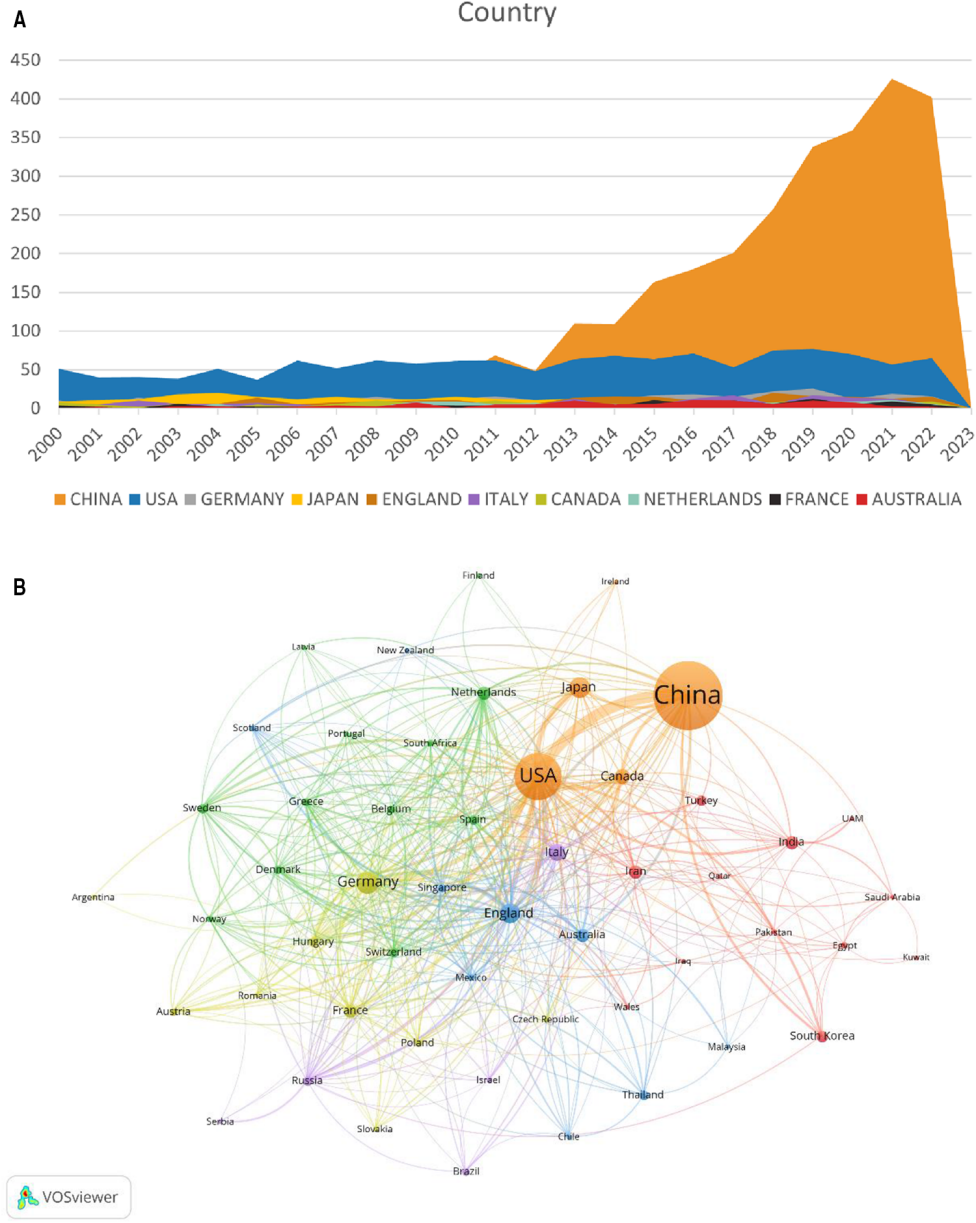
Distribution of countries/regions involved in MIRI. (**A**) The annual number of papers published by the top 10 countries. (**B**) Cooccurrence diagram of countries/regions with more than 5 publications. The size of the nodes corresponds to the publication volume of countries/regions. The lines represent the connections between countries/regions, and their thickness represents the link strength. Different colors represent different clusters.

A total of 81 countries/regions participated in research on MIRI. Table 1 and Figure 3A show the top 10 countries/regions in terms of publication volume, as well as their citation counts, centrality, and annual publication volume. According to the statistics, China (2,845 papers) surpassed the United States (1,325 papers) in publication volume since 2011, followed by Germany (305 papers), Japan (278 papers), and England (233 papers). The United States had the highest citation count (75,508 times), followed by China (56,807 times), England (18,535 times), Germany (15,967 times), and Japan (13,349 times). The citation frequency of other countries was less than 10,000. Centrality of countries/regions is an important indicator of their importance. From the perspective of centrality, the United States, China, England, Italy, and Germany have high centrality and play important roles in this field.

**Table 1:** Top 10 productive countries/regions in the field of MIRI.

Rank	Country/Region	Publications	Citations	Centrality
1	CHINA	2,845	56,807	0.19
2	USA	1,325	75,508	0.31
3	GERMANY	305	15,967	0.13
4	JAPAN	278	13,349	0.01
5	ENGLAND	233	18,535	0.19
6	ITALY	182	9,002	0.16
7	CANADA	135	8,834	0.03
8	NETHERLANDS	117	6,749	0.11
9	FRANCE	109	4,486	0.1
10	AUSTRALIA	108	3,337	0.02

USA, the United States of America.

Figure 3B shows a visualization analysis of 52 countries/regions with publication volume exceeding 5 papers. The size of the nodes corresponds to the publication volume, and the lines represent the connections between countries/regions. The countries/regions are roughly divided into six clusters based on the degree of cooperation, which are represented by different colors. The connections between countries/regions are mainly focused on the cooperation between the US and other countries, including China, Germany and Japan.

### Contribution of institutions

**Figure 4:**
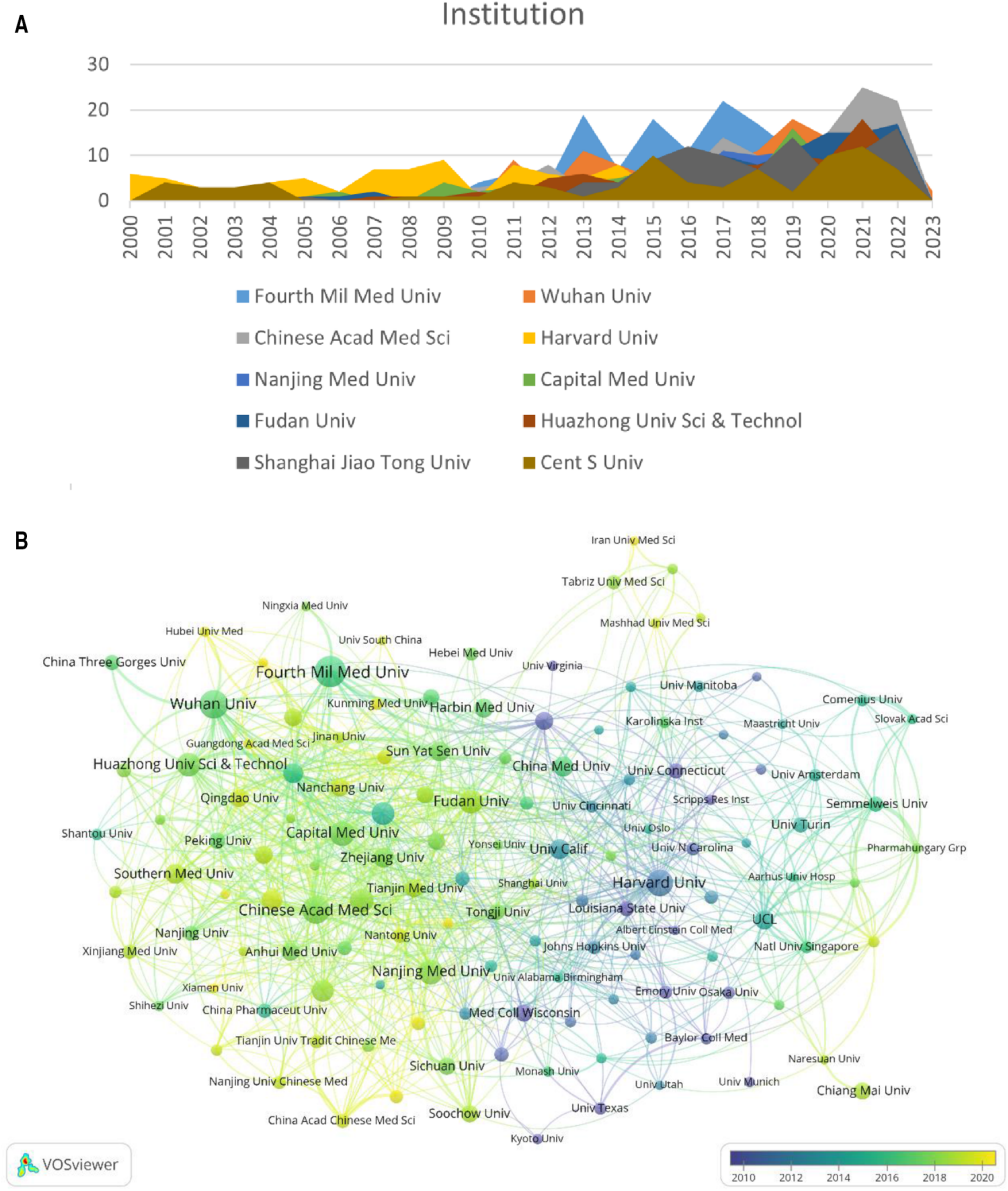

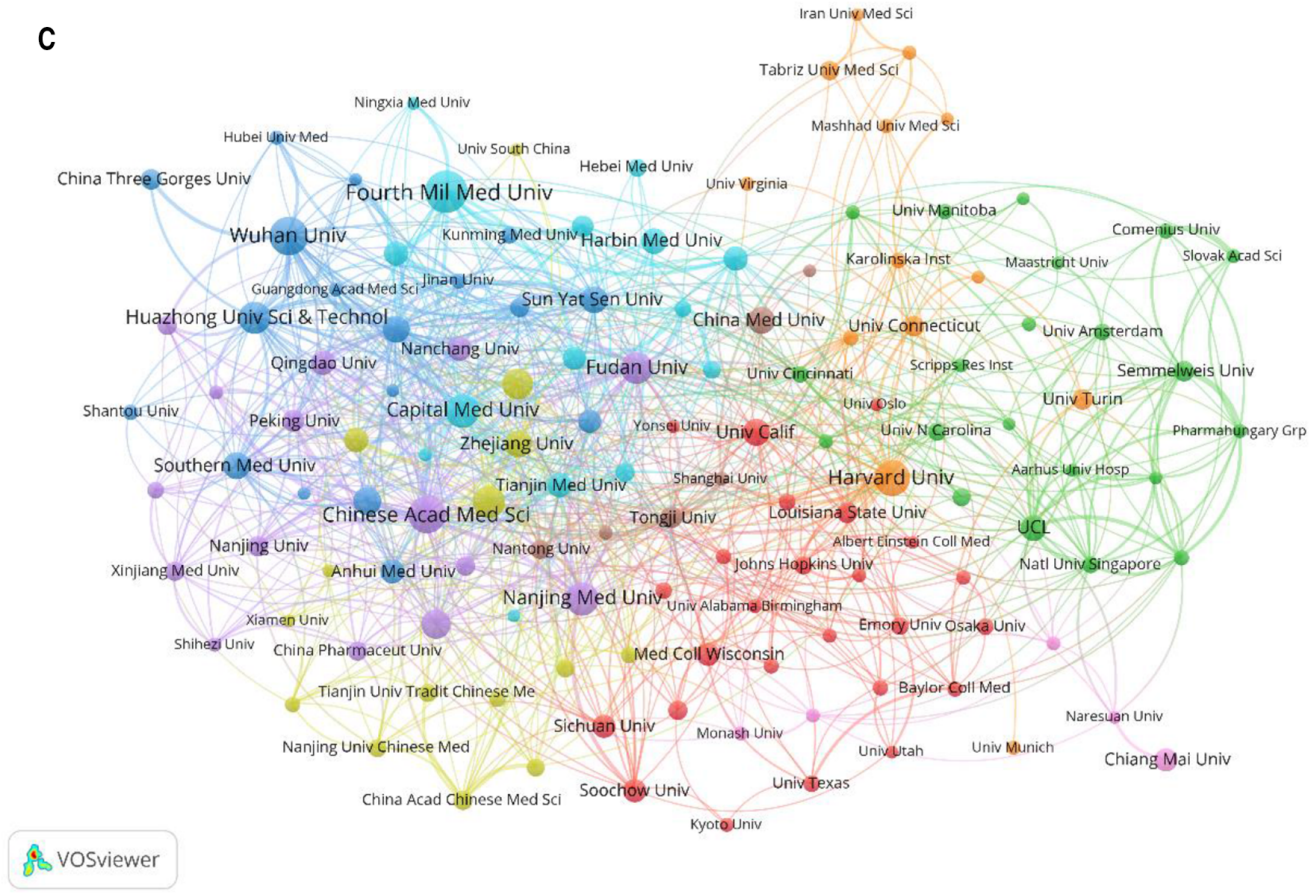
Distribution of institutions involved in MIRI. (**A**) The annual number of papers published by the top 10 institutions. (**B**) The time-overlay map of the cooperation network among the institutions. The color of an item is determined by the average year, where colors range from blue (2000y) to green to yellow (2023y). (**C**) Cooccurrence diagram of institutions with more than 15 publications. The size of the nodes corresponds to the publication volume of institutions. The lines represent the connections between institutions, and their thickness represents the link strength. Different colors represent different clusters.

A total of 3,840 institutions participated in research on MIRI after merging and eliminating meaningless nodes. Statistics and visual analysis were performed on 134 institutions with a publication volume of more than 15. Table 2 shows the top 10 institutions by publication volume, of which 9 are from China, but their centrality is low (<0.1). Harvard University is the only research institution that exceeds 0.1 and has the highest citation volume (7,372 times). It is worth noting that although University College London has only 59 publications, it ranks third in terms of citation volume (6,295 times). According to Figure 4A and Figure 4B, it can be seen that European and American countries, represented by Harvard University with relatively stable publication volume, began research in this area earlier; while Chinese research institutions have shown a significant fluctuating growth trend in the past decade.

**Figure 5:**
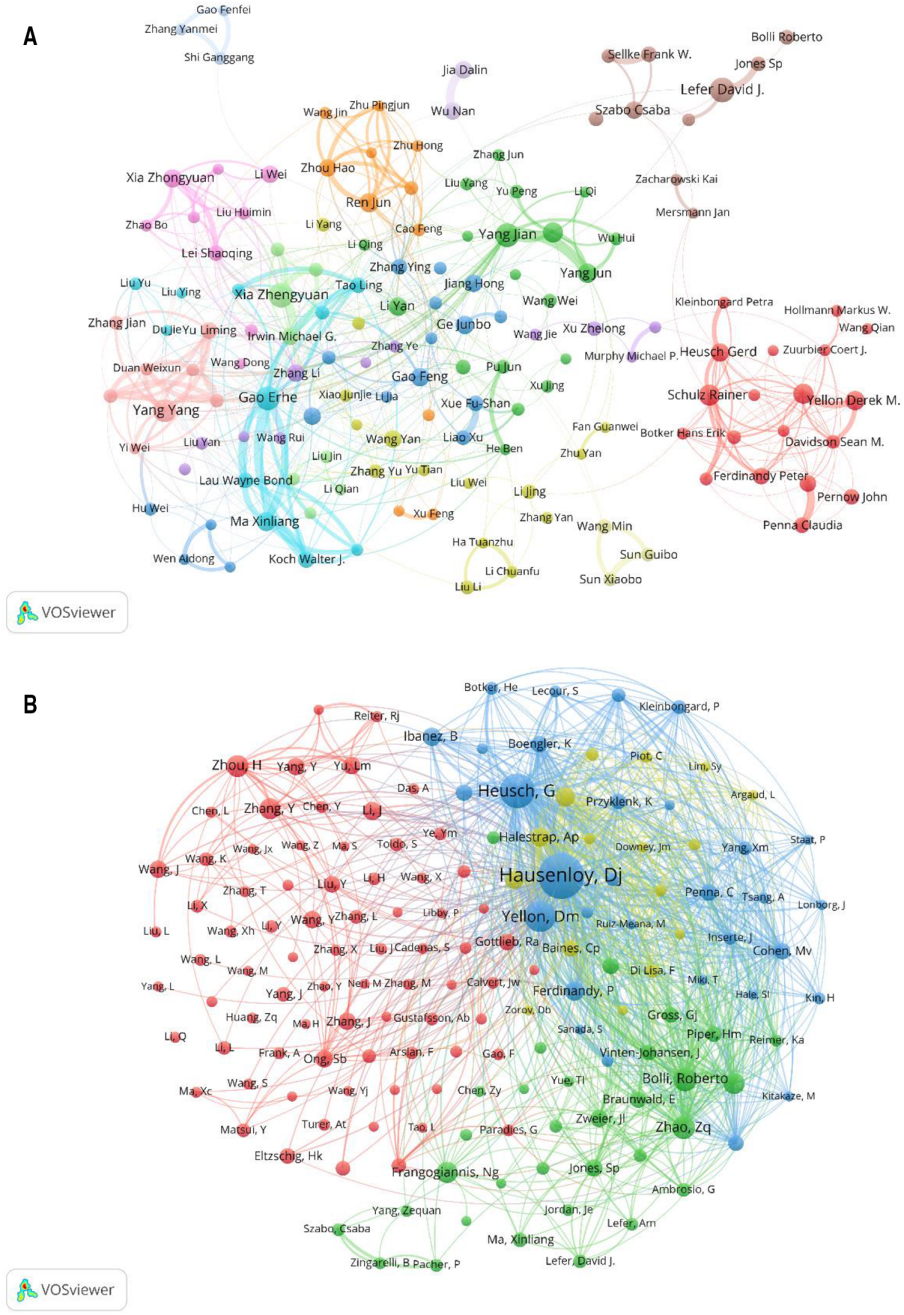
Distribution of authors involved in MIRI. (**A**) Cooccurrence diagram of authors. with more than 10 publications. The size of the nodes corresponds to the publications by the author, and the connections between the nodes reflect the collaboration relationship. Different colors represent different clusters. (**B**) Cooccurrence diagram of co-cited authors. The size of the node in the map represents the total frequency of co-citation. The larger the node, the more frequently it is co-cited.

**Table 2:** Top 10 productive institutions in the field of MIRI.

Rank	Institution	Publications	Citations	Centrality
1	Fourth Mil Med Univ	153	6,427	0.04
2	Wuhan Univ	127	2,969	0.05
3	Chinese Acad Med Sci	115	2,279	0.09
4	Harvard Univ	108	7,372	0.28
5	Nanjing Med Univ	98	2,145	0.03
6	Capital Med Univ	96	1,729	0.04
7	Fudan Univ	91	1,971	0.08
8	Huazhong Univ Sci & Technol	91	1,840	0.06
9	Shanghai Jiao Tong Univ	89	2,461	0.03
10	Cent S Univ	84	1,640	0.02

In this field, the cooperation between institutions is relatively close and international exchanges are frequent. For example, the Fourth Military Medical University has cooperated with other institutions 80 times, especially with Temple University (14 times). In addition, two academic groups have been formed, one represented by Fudan Univ, Chinese Acad Med Sci and Nanjing Med Univ, and the other by Wuhan Univ and Huazhong Univ Sci & Technol, as shown in Figure 4C.

### Authors and co-cited authors

Over the past 20 years, a total of 26,202 authors have participated in research related to MIRI. Among them, 168 authors with more than 10 publications were selected for visualization analysis. The largest number of papers was published by Lefer, David J. and Xia, Zhengyuan (45), followed by Gao, Erhe and Yang, Jian (42). The most co-cited author was Yellon, Derek M. (6,774), followed by Hausenloy, Derek J. (6,601), as shown in Table 3.

**Table 3:** Top 10 productive authors and co-cited authors in the field of MIRI.

Rank	Author	Publication	Country	Institution	Co-cited Author	Citation	Country	Institution
1	Lefer David J.	45	USA	Louisiana State University Health Sciences Center New Orleans	Hausenloy, Derek J.	2,100	ENGLAND	University of London
2	Xia Zhengyuan	45	Hong Kong, China	University of Hong Kong	Heusch, Gerd	1,180	Germany	University of Duisburg Essen
3	Gao Erhe	42	USA	Pennsylvania Commonwealth System of Higher Education	Yellon, Derek M.	944	ENGLAND	University of London
4	Yang Jian	42	China	China Three Gorges University	Bolli Roberto	592	USA	Univ Louisville
5	Yang Yang	38	China	Fourth Mil Med Univ	Zhao Zq	536	USA	Emory Univ
6	Schulz Rainer	33	Germany	Univ Giessen	Zhou, Hao	482	China	Chinese Peoples Liberat Army Gen Hosp
7	Hausenloy Derek J.	31	ENGLAND	University of London	Kloner Ra	468	USA	University of Southern California
8	Zhang Jing	30	China	Fourth Mil Med Univ	Frangogiannis Ng	459	USA	Albert Einstein Coll Med
9	Yellon Derek M.	29	ENGLAND	University of London	Zhang Y	445	China	Peking Univ
10	Ma Xinliang	28	USA	Jefferson University	Ferdinandy, Peter	393	Hungary	Univ Szeged

The collaboration among the authors of MIRI-related literature was displayed in VOSviewer. The same cluster often represents close collaboration and provides information for finding research partners. Several academic groups with relatively fixed collaborations have emerged in this field, as shown in Figure 5A. Furthermore, we can see that academic groups represented by Xia Zhengyuan, Gao Erhe, Yang Jian have more frequently with the outside world, while academic groups represented by Lefer David J. and Schulz Rainer are relatively isolated.

When two or more authors are cited by the same article, a co-citation relationship exists. The size of the node in the map represents the total frequency of co-citation. The larger the node, the more frequently it is co-cited, indicating greater influence in the field. Figure 5B shows that the research hotspots of the authors are highly homogeneous. The authors are mainly divided into 4 clusters: Hausenloy Derek J, Heusch Gerd etc. (blue); Bolli Roberto, Zhao ZQ etc. (green); Zhou Hao, Zhang Y etc. (red); Halestrap AP, Murry CE etc. (yellow).

### Highly co-cited references

Co-citation analysis is a dynamic process that changes over time. It is used to study the internal connections between literature and depict the dynamic structure of scientific development.

**Figure 6:**
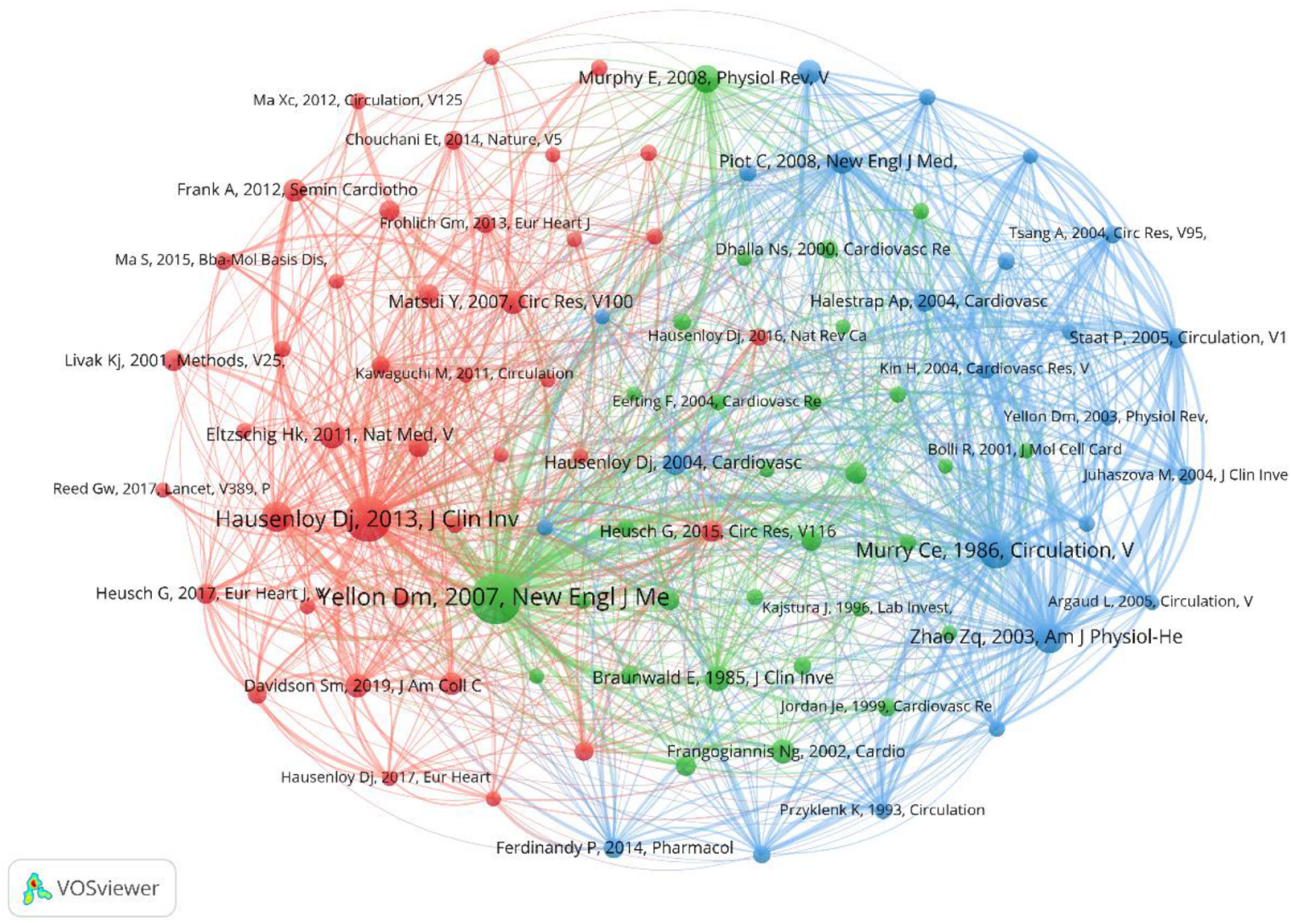
Cooccurrence diagram of references with co-citations exceeding 60 times. The size of the node indicates the cooccurrence frequencies of references, and the link reflects the cooccurrence relationship. The color of the node represents the respective cluster.

The top 10 co-cited papers, totaling 11 papers, are listed in Table 4. In addition, 94 papers with co-citations exceeding 60 times were subjected to visual analysis, where the size of the nodes was proportional to the number of co-citations (Figure 6). The papers were divided into three clusters. The green cluster was led by “Mechanisms of disease: Myocardial reperfusion injury” (Yellon DM, 2007), with the highest number of co-citations (631 times). It mainly described four types of cardiac dysfunction caused by reperfusion injury, and summarizes the reasons for the discrepancies in outcomes of single-target interventions for MIRI in animal models and clinical studies. The study also confirmed new strategies to prevent lethal reperfusion injury by reperfusion injury salvage kinase (RISK) pathway and mitochondrial permeability transition pore (mPTP) (1).

**Table 4:** Top 10 co-cited references.

Rank	Title	Author	Year	Journal	IF
1	Mechanisms of disease: myocardial reperfusion injury	Yellon, Derek M.	2007	NEW ENGLAND JOURNAL OF MEDICINE	176.082
2	Myocardial ischemia-reperfusion injury: a neglected therapeutic target	Hausenloy, Derek J.	2013	JOURNAL OF CLINICAL INVESTIGATION	19.477
3	Preconditioning with ischemia: a delay of lethal cell injury in ischemic myocardium	Murry, Charles E.	1986	CIRCULATION	39.922
4	Inhibition of myocardial injury by ischemic postconditioning during reperfusion: comparison with ischemic preconditioning	Zhao, ZQ	2003	AMERICAN JOURNAL OF PHYSIOLOGY-HEART AND CIRCULATORY PHYSIOLOGY	5.125
5	Evolving Therapies for Myocardial Ischemia/Reperfusion Injury	Ibanez, Borja	2015	JOURNAL OF THE AMERICAN COLLEGE OF CARDIOLOGY	27.206
6	Mechanisms underlying acute protection from cardiac ischemia-reperfusion injury	Murphy, Elizabeth	2008	PHYSIOLOGICAL REVIEWS	46.513
7	Ischemia and reperfusion-from mechanism to translation	Eltzschig, Holger K.	2011	NATURE MEDICINE	87.244
8	Myocardial reperfusion: a double-edged sword?	Braunwald, Eugene	1985	JOURNAL OF CLINICAL INVESTIGATION	19.477
9	New directions for protecting the heart against ischaemia-reperfusion injury: targeting the Reperfusion Injury Salvage Kinase (RISK)-pathway	Hausenloy, Derek J.	2004	CARDIOVASCULAR RESEARCH	14.239
10	Distinct roles of autophagy in the heart during ischemia and reperfusion—roles of AMP-activated protein kinase and Beclin 1 in mediating autophagy	Matsui, Yutaka	2007	CIRCULATION RESEARCH	23.218
10	Effect of cyclosporine on reperfusion injury in acute myocardial infarction	Piot, Christophe	2008	NEW ENGLAND JOURNAL OF MEDICINE	176.082

The red cluster was led by “Myocardial ischemia-reperfusion injury: a neglected therapeutic target” (Hausenloy DJ, 2013), with the highest number of co-citations (529 times). The article identified four recognized forms of MIRI, namely, reperfusion-induced arrhythmias, myocardial stunning, microvascular obstruction (MVO), and lethal myocardial reperfusion injury. It discussed in detail pathological mechanisms such as oxidative stress, calcium overload, pH value correction, mPTP, inflammation, and cell apoptosis, as well as new therapies. However, the article did not affirm the view that MI area would increase with prolonged reperfusion time. In addition, the study confirmed the position of cardiac magnetic resonance (CMR) imaging in the diagnosis and efficacy evaluation of MIRI (17).

The blue cluster was led by “Preconditioning with ischemia: a delay of lethal cell injury in ischemic myocardium” (Murry CE, 1986), followed by “Inhibition of myocardial injury by ischemic postconditioning during reperfusion: comparison with ischemic preconditioning” (Zhao ZQ, 2003) with 322 and 238 co-citations, respectively. The former was proposed by Murry et al. (18) using a canine model, which found that multiple brief ischemic episodes over a period of time could protect the heart from subsequent sustained ischemic injury, thereby introducing the concept of ischemic preconditioning. The latter, proposed by Zhi-Qing Zhao et al. (19), compared the effects of ischemic postconditioning and ischemic preconditioning and demonstrated that both were equally effective in reducing infarct size and protecting endothelial function.

### Distribution of journals

The dual-map overlay of journals reveal the relatives position of the topic of study to the main research disciplines. Each point on the map represents a journal, with the citation graph on the left and the cited graph on the right. The curve represents the validation line, with different colors representing different citation relationships.

**Figure 7:**
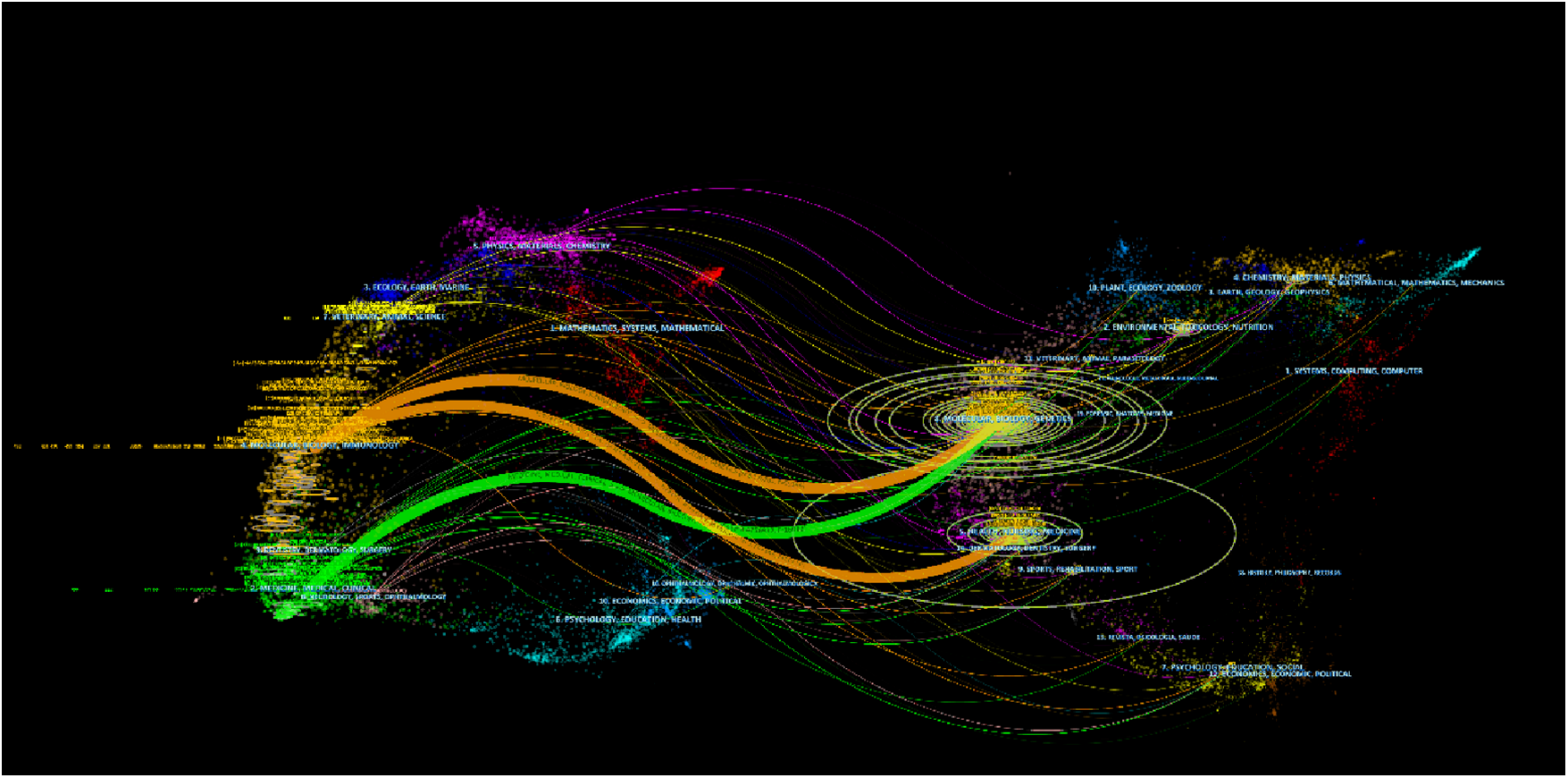
The dual-map overlay of journals. Each node on the map represents a journal, with the citation graph on the left and the cited graph on the right. The curve represents the validation line, with different colors representing different citation relationships.

Figure 7 identifies three main paths, indicating that papers published in the “4 Molecular Biology, Biology, and Immunology” journal primarily reference papers in the fields of “8 Molecular Biology, Biology, and Genetics” and “5 Health, Nursing, and Medicine”. In addition, papers published in journals such as “8 Molecular Biology, Biology, and Genetics” are also commonly cited in papers in the “2 Medicine, Medicine, and Clinical” field. Currently, research on MIRI is mainly focused on clinical and molecular biology aspects.

### Analysis of research hotspots

The keywords summarize the research topic of a paper and can be used to analyze the research hotspots and directions in the field of MIRI. Before visualization, synonyms (e.g., salvia miltiorrhiza and danshen), different spellings (e.g., ischemia, ischemic, and ischaemic), abbreviations (eg, IL-6 and interleukin-6), and singular/plural forms (e.g., arrhythmia and arrhythmias) should be merged. In VOSviewer, the keyword threshold was set at 15, resulting in a total of 142 keywords. The most frequent keyword was “MIRI” (2,918 times), followed by “myocardial ischemia” (734 times) and “apoptosis” (700 times), as shown in Table 5. These keywords can be divided into four different directions: (1) keywords related to risk factors are diabetes mellitus, hyperlipidemia, hypertension, and aging; (2) keywords related to poor prognosis, such as arrhythmia, myocardial stunning, cardiac function, and heart failure; (3) the study of pathological and physiological mechanisms mainly revolves around cell death, oxidative stress, inflammation, endoplasmic reticulum and mitochondria, non-coding RNAs (miRNA, lncRNA), and biomarkers, involving hot signaling pathways such as the PI3K/AKT pathway, Nf-κB pathway, and TLRs signaling pathway; (4) the main treatment-related keywords are cardiac protection, ischemic preconditioning, ischemic postconditioning, melatonin, dexmedetomidine, resveratrol, and Danshen, among others.

**Table 5:** Top 20 keywords in the field of MIRI.

Rank	Keywords	*n*	Rank	Keywords	*n*
1	MIRI	2,918	11	heart	205
2	myocardial ischemia	734	12	ROS	203
3	apoptosis	700	13	diabetes mellitus	162
4	MI	612	14	PI3K/AKT	158
5	cardioprotection	431	15	NO	150
6	oxidative stress	395	16	H/R injury	120
7	inflammation	336	17	antioxidants	112
8	cardiac myocytes	261	18	cardiovascular diseases	105
9	mitochondria	224	19	heart failure	102
10	autophagy	212	20	ER stress	91

**Figure 8:**
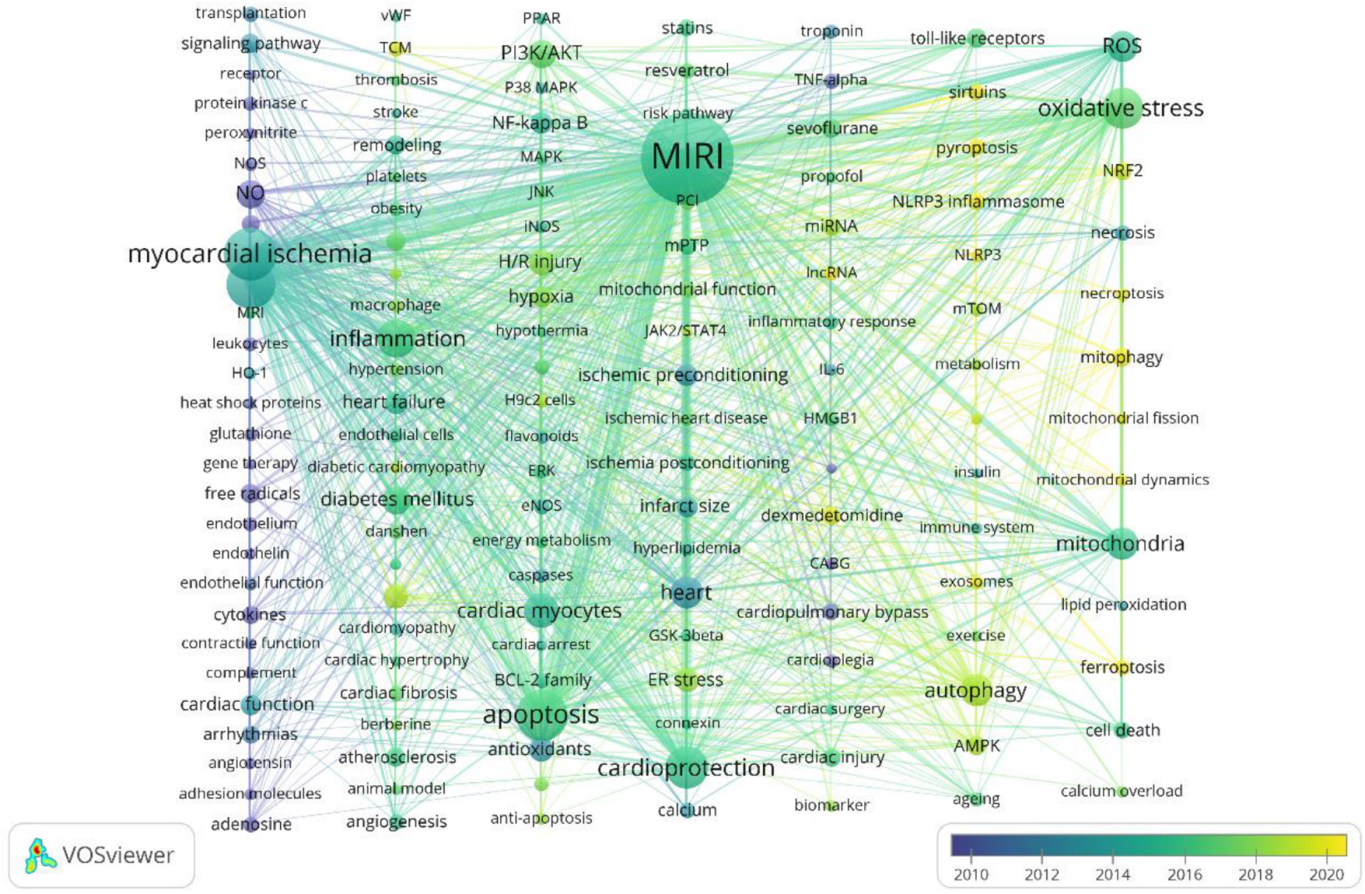
VOSviewer overlay visualization of keywords. Each column represents a cluster, and the color represents the average time. The closer the blue item is, the earlier it appears.

In the overlay visualization of keyword time series (Figure 8), each column represents a cluster, and the color represents the average time. The closer the color is to blue, the more frequently the keyword appeared in the early stage, and the closer to yellow, the more frequently the keyword appeared in recent years, which can reflect the research hotspot in a field to some extent. Keywords such as ferroptosis (2021.472), pyroptosis (2021.095), NLRP3 (2020.4), lncRNA (2020.25), mitochondrial dynamics (2020.2353), exosomes (2019.9643), mitophagy (2019.804), sirtuins (2019.7273), and necroptosis (2019.6522) have been frequently appearing in recent years, indicating that they are hotspots in the field of MIRI in recent years. It is worth noting that traditional Chinese medicine (TCM) (2019.2727) has gradually attracted attention worldwide for its role in treating MIRI. Representative drugs and effective ingredients include danshen, berberine, flavonoids and so on.

## Discussion

### General distribution

In terms of publications, the quantity of papers on myocardial ischemia- reperfusion injury has surpassed 100 papers per year since 2003. Since 2021, it has further increased to over 500 papers per year, indicating that research on MIRI remains a major focus in the cardiovascular field. Among them, the total number of papers published in China is more than 2,850, accounting for more than half of the total publication output. Especially since 2013, the number of publications has rapidly increased, indicating that research on MIRI has received increasing attention from the Chinese in the past decade. However, there is still a problem of insufficient influence. As one of the earliest institutions to begin research on myocardial ischemia reperfusion injury, the United States is another country with more than 1,000 publications, with the highest centrality. Among the top 10 most co-cited authors, 4 are from the United States.

In terms of cooperative relationships, frequent collaborations exist between countries/regions and institutions. For example, European and American academic institutions represented by Harvard University and University College London, and Chinese academic institutions represented by Fudan University and the Chinese Academy of Medical Sciences, all maintaining frequent collaborative relationships with other institutions.

### Hotspots and frontiers

#### In risk factors

In risk factors, the most common keywords related to MIRI are diabetes mellitus, hyperlipidemias, hypertension, and aging, all of which have been reported to be associated with MIRI (20–23). Diabetes mellitus is a common comorbidity in cardiovascular disease patients and increases the risk of cardiovascular disease by 2–4 times compared to non-diabetic patients (24, 25). However, the controversy remains as to whether it increases the susceptibility of the heart to ischemia-reperfusion injury (26). In the co-morbidity study of MIRI and diabetes mellitus, a popular target is AMPK, which has been found to improve cardiovascular complications related to diabetes mellitus by stimulating AMPK. The mechanism mainly involves the downregulation of AMPK in the heart tissue of animals and humans with type 2 diabetes mellitus or metabolic syndrome, leading to energy metabolism disorder, aggravated inflammation, and intensified cellular autophagy, apoptosis, ferroptosis, and necroptosis pathways (27–30).

In addition, MVO as a potentially preexisting risk factor worsens within minutes after reperfusion and persists for at least 1 week (31), resulting in myocardial damage due to inadequate perfusion, known as the “no-reflow” phenomenon (32–34). Approximately 50% of acute MI patients experience ischemia-reperfusion injury in cardiac microvascular endothelial cells (CMECs), which is the main factor leading to the final infarct size and adverse cardiovascular outcomes (35, 36).

#### On the prognosis

On the prognosis, research indicates that reperfusion injury accounts for up to 50% of the final myocardial damage in acute MI (37). Common sequelae of reperfusion injury include heart failure, remodeling, arrhythmias, and myocardial stunning, which are consistent with our survey results.

Acute ST-segment elevation myocardial infarction (STEMI) patients who undergo primary PCI are prone to develop ventricular arrhythmias following ischemia-reperfusion injury, which are usually easily managed or self-terminated (38). The underlying cause may be related to the instability of action potential resulting from the inability of mitochondria to recover or maintain their inner membrane potential after prolonged ischemia (39–41). Moreover, this phenomenon is more common and severe in elderly female rats (42), which may be associated with the decline of estrogen receptors and antioxidant activity in their myocardium, rather than the decrease in serum estrogen levels (43).

Myocardial stunning refers to systolic and diastolic dysfunction in patients with acute myocardial ischemia after reperfusion, and the severity is proportional to the duration of ischemia (17). The mechanism of myocardial stunning is relatively mature. It mainly attributes to the massive formation of reactive oxygen species and calcium overload in cardiomyocytes and microvascular endothelial cells after reperfusion, which leads to endothelial dysfunction and decreased responsiveness to calcium in the excitation-contraction coupling mechanism. However this process is entirely reversible and generally lasts for several hours or days (44–46).

Infarct size is the main determinant of patient prognosis, and MIRI may further increase the infarct size (47, 48). There are two recognized forms of irreversible MIRI: MVO and lethal myocardial reperfusion injury (17, 49, 50). In the early stages of MI, ventricular compensatory mechanisms are activated, and cardiac function remains normal or slightly reduced. As the infarct size expands and non-infarcted areas remodel, the risk of developing heart failure or death remains high (51, 52). A questionnaire survey involving 850 patients showed that 2 out of every 5 patients had heart failure-related quality of life impairment after MI, causing a significant social and economic burden (53).

#### Regarding the mechanisms

Regarding the mechanisms, we found that the research fields with higher output focus on regulated cell death, oxidative stress, inflammatory response, non-coding RNA, as well as mitochondrial and endoplasmic reticulum stress. They form an intertwined association between different pathways that affect MIRI by regulating common pathway molecules. As the main type of cell death during reperfusion, regulated cell death plays an important role in the pathogenesis of MIRI (54). In our VOSviewer visualization, apoptosis has received the most attention. Nevertheless, non-apoptotic forms of regulated cell death, such as ferroptosis, pyroptosis, necroptosis, and autophagy have increasingly received the attention of researchers in recent years. They can operate alone or coexist with other forms of cell death, thereby playing a role in the enlargement of infarct size and the deterioration of heart function caused by ischemia-reperfusion (26, 55). The generation of reactive oxygen species (ROS) is the central pathogenic mechanism of MIRI. Ferroptosis is an iron overload and iron-dependent ROS accumulation process, and its regulatory mechanisms involve multiple signaling pathways and metabolic pathways, especially the glutathione peroxidase 4 (GPX4) axis (10, 26, 56). During myocardial ischemia-reperfusion, the increase in intracellular free iron levels and the decrease in GPX4 activity lead to the massive release of ROS within myocardial cells, causing lipid peroxidation (57–60). In addition, under the chemotactic effect of ROS, neutrophils accumulate in infarcted myocardial tissue several hours after reperfusion, and ferroptosis also promotes this process through Toll-like receptor 4-dependent signaling pathways, triggering harmful inflammatory responses and ultimately leading to cell death (17, 61). Studies have shown that this process mainly occurs during the reperfusion phase of MIRI rather than the ischemic phase (62, 63). It has been found that ferroptosis can be effectively inhibited by ferrostatin 1, liproxstatin 1, iron chelators, and antioxidants during reperfusion, which can protect against myocardial injury, reduce infarct size, and improve cardiac function in acute or chronic MIRI (62, 64–69).

Pyroptosis is a highly inflammatory form of cell death contributing to ischemia-reperfusion injury when overactivated. After reperfusion, the increased levels of calcium ions and ROS lead to the formation of NLRP3 inflammasomes that activate caspases, initiating the pyroptotic pathway (26, 70). On the hand, activated caspase-1/11 can activate Gasdermin D (GSDMD), a pore-forming protein that mediates cell death, increasing cell permeability and resulting in cell lysis typically (71–73). On the other hand, activated caspases cleave IL-1β and IL-18, releasing them outside the cell through GSDMD membrane pores, further triggering inflammation (26, 74). However, Shi et al. (75) found that caspase-11 may be the only pathway to trigger pyroptosis in cardiac myocytes. They also demonstrated that knocking out the GSDMD gene significantly reduced the levels of LDH and IL-18 after hypoxia/reoxygenation, and reduced the area of MI induced by ischemia-reperfusion in mice.

Necrosis has long been considered an uncontrolled form of cell death, but it has been found to occur in a regulated manner as well, known as necroptosis. Necroptosis involves the activation of the RIPK1/RIPK3/MLKL pathway and is recognized as another major programmed cell death type in MIRI now (76, 77). The process involves ischemia and oxidative stress-induced cardiac injury, as follows: the classical necroptotic pathway is usually initiated by the phosphorylation of RIPK1, which further phosphorylates RIPK3. The complex formed by RIPK1 and RIPK3 can induce MLKL oligomerization and translocation to the plasma membrane, leading to Ca2+ or Na+ ion influx and directly forming a pore, releasing damage-associated molecular patterns and causing membrane rupture (78, 79). However, RIPK3-induced myocardial necrosis can also occur independently of RIPK1 (80). When MIRI occurs, RIPK3 can be directly activated. Then through the RIPK3-CaMKII or RIPK3-PGAM5-CypD cascade, promoting the opening of the mPTP and participating in multiple signaling pathways that induce myocardial death (26, 81–84).

Multiple studies prove autophagy is primarily a pro-survival mechanism during short-term ischemia and hypoxia (9, 85). When the supply of oxygen and nutrients to cardiomyocytes is reduced and ATP is depleted quickly, the AMPK/mTOR pathway is activated (86, 87). At this time, cardiomyocytes utilize autophagy to degrade excessive or potentially dangerous cytosolic entities, such as damaged organelles or misfolded proteins, and acquire metabolic substrates to increase ATP production (88, 89). At the same time, an appropriate level of autophagy can reduce ROS production, decrease NLRP3-related inflammatory responses, and decrease other types of cell death, including necroptosis and apoptosis (90). However, research indicates that autophagy plays a dual role in MIRI, depending on the degree of its activation (91, 92). Unlike the ischemic phase, ROS accumulation is deemed the primary factor affecting autophagic flux during the reperfusion phase (93, 94). Elevated levels of ROS during reperfusion cause the opening of the mitochondrial permeability transition pore (MPTP), which promotes ROS release, activates Bnip3 (95–97), and induces the expression of the autophagy-related protein Beclin1 (93, 98). In normal conditions, the anti-apoptotic protein Bcl-2 binds to Beclin1, preventing autophagy. However, this balance could be disrupted by Bnip3, promoting autophagosome formation and increasing the autophagy rate (99, 100), eventually leading to cell death caused by excessive degradation of cellular components (54). Nevertheless, some perspectives propose that ischemia-reperfusion injury is associated with deficiencies in autophagosome-lysosome fusion (88, 101), which lead to cell death by impaired clearance of autophagosomes.

As an entrance to molecular regulators, non-coding RNAs (including miRNAs, lncRNAs, circRNAs) affect cellular function through targeting various molecules in signalling pathways and have been widely studied in cardiovascular disease (102, 103). Among them, miRNAs are the most widely studied ncRNAs. Elevated levels of ROS can cause DNA damage and regulate miRNAs, which can negatively regulate gene expression by inducing mRNA degradation or inhibiting their translation (104, 105). Previous studies have shown that during MIRI, miR-29c and miR-125a are significantly downregulated (106, 107), while miR-135b-3p is upregulated (108), playing roles in promoting autophagy and ferroptosis, respectively. *In vivo*/*in vitro* experiments showed that modulation of miR-1, miR-126, miR-140-3p, miR-214-5p, miR-125b and miR-24 could exert anti-apoptotic effects (109–114). Regulation of miR-133a, miR-15 exert anti-apoptotic effects (115–117). Moreover, miRNAs can bind to several mRNA molecules, allowing them to play multiple cellular functions. For example, miR-29b binds to PTEN. Its overexpression can reduce PTEN expression level and increased the protein levels of p-Akt/Akt and p-eNOS/eNOS, thereby exerting Anti-oxidative stress, Anti-inflammatory and Anti-apoptosis effects (118). MiR-125a-5p targets KLF13, TGFBR1, and DAAM1, promoting M2 macrophage polarization, inhibiting fibroblast proliferation and activation, and promoting angiogenesis, subsequently improving myocardial cell apoptosis and inflammation (107). Table 6 provides a summary of some important miRNAs. Table 6 provides a summary of some important miRNAs.

**Table 6:** The regulatory role of MicroRNAs in MIRI.

MicroRNAs	Species	Expression	Targeted genes	Mechanism	Refs.
miR-1	rat	↓	Hsp90aa1	Pro-apoptosis	(109)
miR-125a	rat/H9c2	↓	DRAM2	Anti-oxidative stress, Anti-autophagy	(107)
miR-125a-5p	mice	↓	KLF13, TGFBR1, DAAM1	Anti-inflammatory, Anti-apoptosis	(119)
miR-125b	rat	↓	SIRT7	Anti-apoptosis	(113)
miR-126	rat	↑	ERRFI1	Anti-apoptosis	(110)
miR-128	mice/H9c2	↓	Plk2	Anti-apoptosis	(120)
miR-128	rat/H9c2	↓	TXNIP	Anti-oxidative stress, Anti-apoptosis	(121)
miR-128-1-5p	rat/H9c2	↓	Gadd45g	Anti-apoptosis	(122)
miR-129	rat/H9c2	↓	PTEN	Anti-apoptosis	(123)
miR-129	cardiomyocytes	↓	TLR4	Anti-inflammatory	(124)
miR-129-5p	H9c2	↓	TRPM7	Anti-inflammatory, Anti-apoptosis	(125)
miR-129-5p	rat	↑	HMGB1	Anti-apoptosis	(126)
miR-133a	rat/H9c2	↓	ELAVL1	Anti-pyroptosis	(116)
miR-133a	rat/H9c2	↓	IGF1R	Anti-apoptosis	(115)
miR-135b-3p	rat/H9c2	↑	GPX4	Pro-Ferroptosis	(108)
miR-138	mice	↓	EGR1	Anti-inflammatory	(127)
miR-138-5p	mice	↓	Ltb4r1	Anti-inflammatory	(128)
miR-140	mice	↓	YES1	Anti-apoptosis	(129)
miR-140-3p	H9c2	↓	PTEN	Anti-oxidative stress, Anti-apoptosis	(111)
miR-15b-5p	rat/H9c2	↑	Sirt3	Anti-pyroptosis	(117)
miR-155-5p	mice	↑	JAK2/STAT1	Pro-inflammation	(130)
miR-155-5p	mice	↑	NEDD4	Pro-apoptosis	(131)
miR-181a-5p	cardiomyocytes	↑	ADCY1	Pro-pyroptosis	(132)
miR-182-5p	rat/H9c2	↑	STK17A	Pro-oxidative stress	(133)
miR-21	mice	↓	SPP1	Anti-oxidative stress, Anti-inflammatory, Anti-apoptosis	(134)
miR-214-5p	mice	↓	FASLG	Anti-apoptosis	(112)
miR-24	rat/H9c2	↑	Keap1	Anti-apoptosis	(114)
miR-29b	rat/H9c2	↓	PTEN	Anti-oxidative stress, Anti-inflammatory, Anti-apoptosis	(118)
miR-29b-3p	rat/H9c2	↓	HMCN1	Anti-oxidative stress, Anti-fibrosis, Anti-apoptosis	(135)
miR-29c	mice	↓	PTEN	Anti-autophagy	(106)
miR-30b	mice	↓	CypD	Anti-necrosis	(136)
miR-486	mice	↓	PTEN, FoxO1	Anti-apoptosis	(137)

#### Cardioprotection

MI commonly causes two processes of myocardial injury, the first occurring during ischemia and the second possibly after reperfusion. The following treatment strategies can be summarized in light of these two processes. The first is mechanical ischemic conditioning, including brief ischemia-reperfusion cycles in the heart or tissues away from the heart, which can be achieved by ischemic preconditioning or postconditioning methods (138–141).

The second strategy involves drug therapy proven to protect the myocardium. For patients with pre-existing coronary artery disease, long-term and standardized treatment has been shown to effectively prevent major adverse cardiovascular events (MACE). Such as aspirin and ticagrelor, which can prevent reperfusion injury when given before reperfusion and effectively limit the area of MI (142, 143). The protective effect of simvastatin on contractile function in acute MIRI models may be related to the inhibition of the RhoA/ROCK pathway. Research shows SGLT2 inhibitors can alleviate the damage of MI in diabetic and non-diabetic hearts (144–146), reduce MIRI by inhibiting cardiomyocyte autophagy and protecting mitochondrial function, and reduce cardiovascular mortality and heart failure (HF) rehospitalization rate of patients after myocardial ischemia-reperfusion by targeting multiple pathways (26, 146–148). Nicorandil is widely used in the treatment of coronary heart disease and has a dual effect as a nitric oxide (NO) donor and increases cell membrane permeability to potassium ions. It has been shown to alleviate oxidative stress, inflammation, and apoptosis induced by ischemia-reperfusion (149, 150). A recent randomized double-blind controlled trial showed that nicorandil administered before primary PCI could improve the myocardial perfusion grade and increase the ejection fraction, and reduce myocardial infarct size in patients with ST-segment elevation MI (151). In addition, the soluble guanylate cyclase (sGC) stimulator vericiguat has been shown to reduce MIRI by improving microcirculation (152). Moreover, there are increasing studies and reports on the use of single or compound traditional Chinese medicine in the prevention and treatment of MIRI (153, 154). Resveratrol can reduce oxidative stress levels, Fe2+ content and inhibit ferroptosis induced by ischemia-reperfusion (155). Yang et al. (156) first reported that neocryptotanshinone can promote autophagolysosome clearance of protein aggregates via the ERK1/2-Nrf2-LAMP2 pathway, exerting therapeutic advantages for MIRI. Other Chinese medicines and effective ingredients, such as Madder, Calenduloside E, and Tubeimoside I, can also reduce infarct size and alleviate MIRI through different mechanisms, such as reducing inflammation, oxidative stress, or inhibiting cell death (157–159).

Thirdly, with the advancement of technology, research on novel therapeutic methods such as nanomedicine has gradually been carried out. Compared to free drugs, nanomedicine has better therapeutic effects and safety, attributed to its multifunctional carrier selection, targeted and controlled drug release, and improved bioavailability (160). Currently, common nanocarriers include liposomes (161–163), polymer nanoparticles (164, 165), inorganic nanoparticles (166) and extracellular vesicles (167–169). Carvedilol, a nonselective β-blocker, was encapsulated into platelet membrane vesicles (PMVs). Targeted administration of PMVs@Carvedilol may be a promising treatment for myocardial reperfusion injury, as it significantly improves postinjury cardiac function and increases drug utilization compared to other delivery methods (170). MicroRNAs use exosomes as a carrier to enable cell-to-cell communication (171). As previously mentioned, MIRI is often accompanied by abnormal expression of miRNAs. Enrichment of specific miRNAs by mesenchymal stem cell-secreted extracellular vesicles has shown promising results in regulating miRNA levels in cardiomyocytes in various preclinical experiments, making it a potential therapeutic approach (102, 172). It is worth noting that some miRNAs are dysregulated in multiple cardiovascular diseases. Therefore, selecting miRNAs that are dysregulated throughout the entire disease process leading to MIRI may have a stronger therapeutic effect. At the same time, it is essential to consider the safe and effective translation of preclinical experiments to clinical practice.

## Limitations

Firstly, this study's bibliometric analysis only includes papers in the WoSCC database, while other databases such as PubMed, Cochrane library, and Google Scholar are excluded. However, it should be noted that WoSCC is widely recognized as one of the most authoritative scientific literature search platforms, covering the vast majority of research on MIRI and still maintaining a certain degree of representativeness. Secondly, papers published in recent years are rarely cited, which may lead to the omission of some recently published papers with significant contributions when analyzing highly co-cited papers, indicating the necessity of updating future research. In addition, changing job positions or using different names within the same institution during an author's career can also have a significant impact on research results.

## Conclusion

Using information visualization techniques, we have attempted to elucidate the research progress, hot topics, and frontiers in MIRI over the past two decades. Although the annual publication output in China has far exceeded that in the United States in recent years, its academic influence is far behind. In addition, we have identified scholars, institutions, and representative literature that play important roles in this field. Keyword analysis shows that the main research direction is the pathogenesis of MIRI and corresponding protective strategies, with ferroptosis and pyroptosis as the latest hot topics.
